# Differentiation of *S. chartarum* (Ehrenb.) *S. Hughes* Chemotypes A and S via FT-IR Spectroscopy

**DOI:** 10.1007/s11046-020-00495-0

**Published:** 2020-10-10

**Authors:** Julia Ekruth, Christoph Gottschalk, Sebastian Ulrich, Manfred Gareis, Karin Schwaiger

**Affiliations:** 1 Chair of Food Safety, Faculty of Veterinary Medicine, Ludwig-Maximilians-University Munich, Schoenleutnerstr. 8, 85764 Oberschleissheim, Germany; 2 Bacteriology and Mycology, Institute for Infectious Diseases and Zoonoses, Department of Veterinary Science, Faculty of Veterinary Medicine, Ludwig-Maximilians-University Munich, Veterinaerstr. 13, 80539 Munich, Germany

**Keywords:** *Stachybotrys chartarum*, Macrocyclic trichothecenes, Triplex PCR, Fourier-transform-infrared spectroscopy

## Abstract

*Stachybotrys *(*S.*)* chartarum* is a cellulolytic mould with the ability to produce highly cytotoxic macrocyclic trichothecenes. Two chemotypes are defined according to their ability to produce either atranones or satratoxins. *S. chartarum* has been well known as the causative agent of the lethal disease stachybotryotoxicosis in horses. Further investigations revealed that this disease is strictly correlated with the presence of macrocyclic trichothecenes. Furthermore, their occurrence in water-damaged buildings has been linked to adverse health effects such as the sick building syndrome. As the chemotypes cannot be characterized via phenotypic criteria, different methods such as PCR, MALDI–TOF MS, LC–MS/MS, thin-layer chromatography and cytotoxicity assays have been used so far. Fourier-transform-infrared spectroscopy (FT-IR) is commonly used for the differentiation of bacteria and yeasts, but this technique is also applicable to filamentous fungi. Hence, this study aimed at evaluating to which extent a reliable differentiation of *S. chartarum* chemotypes A and S is possible. Besides, another objective was to verify if the recently introduced third genotype of *S. chartarum* can be identified. Therefore, 28 strains including the two chemotypes and the third genotype H were cultivated on malt extract agar (MEA) and potato dextrose agar in three biological replicates. Each sample was applied to FT-IR measurements on day 7, 14 and 21 of cultivation. In this study, we achieved a distinction of the chemotypes A and S via FT-IR spectroscopy after incubation for 7 days on MEA. In terms of genotype differentiation, the PCR detecting satratoxin- and atranone-gene clusters remained the only applicable method.

**Electronic supplementary material:**

The online version of this article (10.1007/s11046-020-00495-0) contains supplementary material, which is available to authorized users.

## Introduction

*Stachybotrys* is a dematiaceous mould comprising 75 species [1, 2]. Due to its cellulolytic activity, it can mostly be isolated from hay, straw, damp building materials (e. g., gypsum board, wallpaper, wood) and culinary herbs [3–8]. It has firstly attracted scientific interest after numerous horses of the former Soviet military died after the ingestion of feed which was highly contaminated with this black mould [9]. This lethal disease was named stachybotrytoxicosis and further investigations identified highly cytotoxic macrocyclic trichothecenes as the actual causative agents [9, 10]. In terms of secondary metabolites, *S. chartarum* is also known to produce atranones and phenylspirodrimanes [11, 12]. The macrocyclic trichothecenes are suspected to cause severe health effects in humans as their occurrence has been linked to cases of sick building syndrome, haemorrhages in infants and other adverse health effects [10, 13–18]. *S. chartarum* is commonly divided into two chemotypes according to their ability to produce atranones (chemotype A) or satratoxins (chemotype S) [11]. In a recently published study, another nomenclature dividing *S. chartarum* isolates into three genotypes was proposed [19]. FT-IR spectroscopy has been successfully used for the identification and characterization of bacteria and yeasts as well as for the differentiation of moulds such as *Fusarium* spp., *Penicillium* spp., *Aspergillus* spp. and many others [20–26]. However, neither *S. chartarum* nor any other species of *Stachybotrys* have been part of FT-IR spectroscopy studies. An IR-spectrometer emits infrared radiation which penetrates the sample and the intensity of the infrared radiation is measured before and after passing through the sample. The intensity of transmission depends on the quantity and quality of the molecular bonds in the specific sample. In the following, the interferogram is converted into a unique infrared spectrum via Fourier transformation [27]. Finally, the spectral data are converted into dendrograms and can be evaluated with the help of hierarchical cluster analysis based on the Euclidean distance [28].

This study aimed at the differentiation of *S. chartarum* chemotypes via FT-IR spectroscopy.

All the selected strains were initially characterized via PCR and then applied to FT-IR spectroscopic analysis. With this technology, the chemotypes A and S could be successfully distinguished after incubation for 7 days on malt extract agar (MEA). Furthermore, the strains were also incubated on potato dextrose agar (PDA) to evaluate if one of the media was more suitable for reliable results.

## Materials and Methods

### Fungal Isolates and Culture Conditions

In total, 28 strains of *S. chartarum* were examined in this study (Table 1). The isolates are divided into three sets: nine strains of chemotype A, nine strains of chemotype S and ten strains of genotype H. Among these, five reference strains were purchased from CBS (Westerdijk Fungal Biodiversity Institute, The Netherlands), IBT (Culture Collection of Fungi, Denmark) and ATCC (American Type Culture Collection). The remaining 23 strains are field isolates and were selected from the in-house culture collection already characterized by different methods (MALDI–TOF MS, sequencing (tri5-fragment) and mycotoxin production by LC–MS/MS). All strains were either stored as lyophilisates or in 20% glycerol as single-spore isolates.

**Table 1:** Strains and characteristics of *Stachybotrys* (*S*.) *chartarum* (*n* = 28, in-house culture collection and reference strains) examined in the present study

ID	Source	Isolated From	Triplex PCR-Typing^b^	Macrocyclic Trichothecenes^c^
S 1074	FSM	Wallpaper	A	−
S 1244	FSM	Gypsum board	A	−
S 1286	FSM	Gypsum board	A	−
S 1353	FSM	Wallpaper	A	−
S 1362	FSM	Wallpaper	A	−
S 1378	FSM	Wallpaper	A	−
S 1431	FSM	Plasterboard	A	−
S 1432	FSM	Plasterboard	A	−
S 1433	FSM	Plasterboard	A	−
IBT 40288^d^	IBT	Building material	A	−
R 07^a,d^	IBT (IBT 40,293)	Building material	S	+
R 24^a^	ATCC (ATCC 34,916)	Oats	S	+
S 1114^a^	MRI (R 03)	Plasterboard	S	+
S 1493/1	FSM	Air sample	S	+
S 35 It	FSM	Straw	S	+
S BB2	FSM	Savoury	S	+
S BO1a	FSM	Oregano	S	+
S BO1b	FSM	Oregano	S	+
S BO2	FSM	Oregano	S	+
IBT 7711^d^	IBT	Building material	S	+
S 1077	FSM	Plasterboard	H	−
S 1285	FSM	Gypsum board	H	−
S 1333	FSM	Gypsum board	H	−
S 1334	FSM	Gypsum board	H	−
S 1335	FSM	Gypsum board	H	−
S 1341	FSM	Wallpaper	H	−
S 29	FSM	Straw	H	−
S 41	FSM	Unknown	H	−
S 42^a^	IBT (HMRB 10)	Unknown	H	−
S 43^a^	CBS (CBS 324.65)	Textile	H	−

*CBS* Westerdijk Fungal Biodiversity Institute, Utrecht, The Netherlands; *FSM* Chair of Food Safety Ludwig-Maximilians-University, Munich, Germany; *IBT* Culture Collection of Fungi, Danish Technical University, Lyngby, Denmark; *ATCC* American Type Culture Collection, Manassas, USA, *MRI* Max Rubner Institut, Kulmbach, Germany

^a^Reference strains

^b^Method according to Ulrich et al. [19]

^c^Prior analyses (Gareis M. Characterization of *Stachybotrys chartarum* strains of the in-house culture collection in terms of toxin production by LC–MS/MS. Personal Communication, 2020.)

^d^Used as positive control for PCR identification

For all identification and characterization steps, the cultures were grown on malt extract agar (MEA, ready-to-use mix, 48.0 g/L, Merck Darmstadt, Germany) and potato dextrose agar (PDA, ready-to-use mix 39.0 g/L, VWR Darmstadt, Germany). Culture media were sterilized at 121 °C for 15 min before use. Working cultures were grown as three-point isolates for 21 days at 25° C and 95% relative humidity. Each strain was cultivated in three biological replicates on both media. In total, six cultures per isolate were analysed with the methods explained below. For the preparation of liquid cultures for FT-IR spectroscopy measurement, malt extract broth (MEB, ready-to-use mix, 17.0 g/L, Merck Darmstadt, Germany) and potato dextrose broth (PDB, ready-to-use mix, 24.0 g/L, VWR Darmstadt, Germany) were used. A photograph of each isolate has been taken, and to confirm the determination of the prevailing genotype, the PCR based on the truncated satratoxin-gene clusters has been carried out as described previously [19].

### Isolation of Fungal DNA and PCR Products

For the isolation of fungal DNA, mycelia of 7-day-old cultures were transferred to an Eppendorf tube (Eppendorf, Hamburg, Germany) with 1000 µL ethanol. The extraction of DNA was performed with the NucleoSpin^®^ Plant II-Kit (Macherey Nagel, Düren, Germany) and a slightly modified instruction as described below. The samples were centrifuged for 5 min at 14,500 rpm (Mini Spin^®^ Plus, Eppendorf, Hamburg, Germany, 12-well rotor, 17,661 rcf) and the supernatant was removed; this step was repeated once. In order to achieve a better cleavage of cells, 150 mg of glass beads (diameter 1 mm, Bio-Spec Products, Karlsruhe, Germany) was added to the 400 µL Lysis buffer and the isolates were treated with a TissueLyser (Qiagen, Hilden, Germany) for 30 s (frequency: 30/sec) [7]. Ten microlitres diluted RNase (NucleoSpin^®^ Plant II-Kit) and 5 µL protein kinase K (20 mg/mL, recombinant PCR grade, Roche, Basel, Switzerland) were added and the samples then incubated for 60 min at 65 °C (Thermomixer Comfort, Eppendorf, Hamburg, Germany). The liquid samples were transferred to a new 1.5-mL reaction tube and after adding of 100 µL chloroform (Merck, Darmstadt, Germany) mixed for 20 s and centrifuged for further 15 min at 14,500 rpm. The supernatant was then gently transferred to a collection tube with a violet filter (NucleoSpin^®^ Plant II-Kit) and centrifuged for 2 min at 11,000 rpm (10,164 rcf). In the following step, 450 µL PC buffer was added. The samples were transferred to a new collection tube with a green filter (NucleoSpin^®^ Plant II-Kit) and centrifuged for 1 min at 11,000 rpm. The green filter was washed with 400 µL washing buffer PW1 (centrifugation for 1 min at 11,000 rpm). The last two washing steps were carried out with the washing buffer PW2 (600 µL and 1 min at 11,000 rpm, 300 µL and 3 min at 11,000 rpm). The filter was transferred to a new 1.5 mL reaction tube. Fifty microlitres elution buffer PE was added to the filter with the bound DNA and the samples were incubated for 5 min at 70 °C followed by a final centrifugation step (1 min at 11,000 rpm). The elution step was repeated once. The DNA extracts were diluted with sterilized water to a final concentration of approximately 10 ng/mL. The concentrations were determined with a NanoDrop 1000 spectrophotometer (PeQlab, Erlangen, Germany). Until further analysis, the DNA extracts were stored at −20 °C.

### PCR Analysis of Satratoxin and Atranone Cluster Genes

The PCR was carried out using two pairs of primers (based on the genome sequences of the macrocyclic trichothecene producing strains IBT 40293, IBT 7711 and the atranone producing strain IBT 40288) as described in an earlier publication [19]. The master mix for 25 µL reactions consisted of the following components: 5 µL Green GoTaq Flexi Buffer (Promega, Mannheim, Germany), 2 µL MgCl_2_ (0.25 mol/L, Promega, Mannheim, Germany), 2.5 µL dNTP (0.2 mol/L, Promega, Mannheim, Germany), 2 µL of each primer (0.05 mol/L, Eurofins, Ebersberg, Germany), 0.25 µL GoTaq G2 Flexi DNA polymerase (5 u/µL, Promega, Mannheim, Germany), sterile demineralized water up to 24 µL. One microliter of genomic DNA (approximately 10 ng/µL) of the tested strains was added to the master mix. The PCRs were run in a Bio-Rad iCycler (Bio-Rad, Munich, Germany) using the following temperature protocol: initial melting at 95 °C, melting 30 s at 95 °C, annealing at 58 °C for 20 s, followed by elongation at 72 °C for 30 s. The cycle of melting, annealing and elongation has been carried out thirty times. The final extension was carried out at 72 °C for 5 min. For final identification, the amplification products were separated on a 1.5% (w/v) agarose gel (Bio-Rad, Munich, Germany) in a TAE buffer (5%, Roth Karlsruhe, Germany). To mark the molecular weight, a 100 bp gene marker was used (HyperLadder, Bioline, Luckenwalde, Germany). Ethidium bromide (0.5 µg/mL, VWR, Darmstadt, Germany) was used for visualization. The gel was inspected in a UV chamber (Bio-Rad, Munich, Germany) and documented with a GelDoc software.

### Sample Preparation for FT-IR Spectroscopy

Mycelia and spores were taken from the working cultures after 7, 14 and 21 days of incubation with a sterile swab (dipped in the prevailing broth) and transferred into centrifuge tubes (15.0 mL Greiner Bio-One, Frickenhausen, Germany) filled with 12.5 mL MEB or PDB to leave enough space for the mycelia to grow. The samples were incubated at room temperature on a rotary shaker for 4 days. On the 4th day of incubation, the tubes were put vertically for 5 min in order to let mycelia pellets settle to the bottom of the tube. The mycelia free broth was gently poured off. 1500 µL of the liquid broth with mycelia pellets was transferred with a pipette (Eppendorf Reference 100–1000 µL, Eppendorf Hamburg, Germany) to a 1.5 mL Eppendorf tube and centrifuged for 5 min at 13,500 rpm (Labogene, Allerød, Denmark, 12-well rotor, corresponds to 18,371 rcf). The supernatant was removed, and the step was repeated with 800 µL of liquid broth. After another centrifugation step, the supernatant was removed again. 1000 µL sterile aqua bidest was added and vortexed for one minute. The samples were centrifuged at 13,500 rpm for 5 min and the liquid was removed. This washing step was repeated twice. Fifty microlitres of ethanol (70%) and four sterile metal beads (Bruker Daltonik GmbH, Germany) were added. To achieve a better cleavage of cells, the samples were treated with a TissueLyser (Qiagen, Hilden, Germany) for 30 s at 30/s. Afterwards, the samples were treated with the short spin function to settle mycelia in the reaction tube and to avoid too much material in the lid. In order to reduce the surface tension, 50 µL sterile aqua bidest was added. The samples were vortexed for 30 s and 15 µL of each sample was spotted on a 96-well microtiter plate (Bruker Daltonik GmbH, Bremen, Germany). The microtiter plate was dried at 37 °C in an incubator until all technical replicates were entirely dried and applied to analysis. The instructions are also displayed as a flow chart in Online Resource 1.

### Quality Check of The Sample Material

Before each measurement, the applied sample material is evaluated regarding different criteria (e.g. absorption, signal-to-noise ratio). The quality check is obligatory and cannot be skipped. The spectral data can only be generated if this check is passed.

### FT-IR Spectroscopy Measurement and Evaluation

FT-IR-spectroscopic measurements were taken with an IR Biotyper spectrometer (Bruker Daltonik GmbH, Bremen, Germany) following the Bruker Daltonik User’s manual for FT-IR spectroscopy (2017) except for the sample preparation protocol. For each strain, 6 technical replicates were prepared for measurement. The analysis of the generated data was performed with the Biotyper software (Version 1.5.0.90, Bruker Daltonik GmbH, Bremen, Germany) and its automated settings. The spectra were recorded up to 1500 cm^−1^ with a spectral resolution of 3 cm^−1^ and an aperture of 10 mm. For each spectrum, 64 scans were averaged. The spectral data were automatically transformed into dendrograms using the average mean spectra method to maintain simplicity in Figs. 4 and 3 [28].

### Statistical Data Analysis

The statistical data analysis was carried out using the SPSS software (version 26.0). In order to determine the accordance between the genotype identification via PCR and the classification via FT-IR spectroscopy, the Cohen’s Kappa values were calculated. The categorization of the Cohen’s Kappa value by Landis, Koch [29] was applied to the statistical data as follows:< 0.00 = poor; 0.00–0.20 = slight; 0.21–0.40 = fair; 0.41–0.60 = moderate; 0.61–0.80 = substantial; 0.81–1.00 = almost perfect accordance. Besides, the statistical significance was calculated and is given as *p* value. The results of the classification were interpreted as statistically relevant if the *p* value was below 0.05. The corresponding results for each measurement are depicted in the description of Figs. 4 and 3.

## Results

This study aimed at the differentiation of the *S. chartarum* chemotypes. Therefore, 28 strains of *S. chartarum* were initially characterized via PCR to identify their satratoxin- and atranone-gene clusters [19]. Moreover, the optimized sample preparation protocol was applied to these strains for their differentiation via FT-IR measurements. The results of the identification via PCR were compared with the chemotype characterization via FT-IR spectroscopy. The accordance of the results was defined with the help of Cohen’s Kappa values [29].

### Identification of S. chartarum Isolates with Triplex PCR

**Fig. 1:**
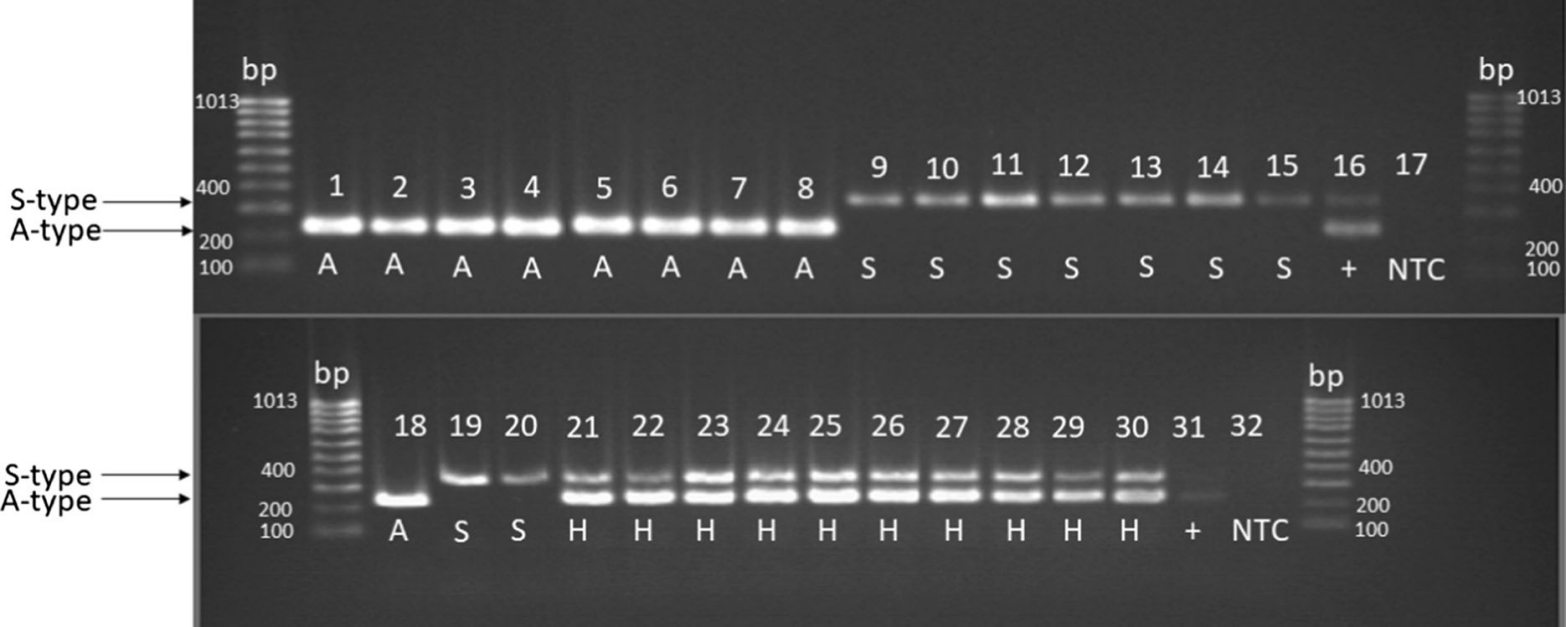
Agarose gel (1.5%) stained with ethidium bromide (0.5 µg/mL) for visualization of the results of triplex PCR for the identification of atranone (chemotype A: 230 bp)- and satratoxin (Chemotype S: 346 bp)-gene clusters in *S. chartarum*; in genotype H isolates, both clusters are detectable: 1 = S 1074, 2 = S 1244, 3 = S 1286, 4 = S 1353, 5 = S 1378, 6 = S 1431, 7 = S 1432, 8 = S 1433, 9 = S 1114, 10 = S 1493/1, 11 = S 35 It, 12 = R 07, 13 = R 24, 14 = S BO1a, 15 = S BO1b, 16 = positive control^a^, 17 = non-template control, 18 = S 1362, 19 = S BO2, 20 = S BB2, 21 = S 1077, 22 = S 1285, 23 = S 1333, 24 = S 1334, 25 = S 1335, 26 = S 1341, 27 = S 29, 28 = S 41, 29 = S 42, 30 = S 43, 31 = positive control^a^, 32 = non-template control a = IBT 40293, IBT 7711 and IBT 40288

**Fig. 2:**
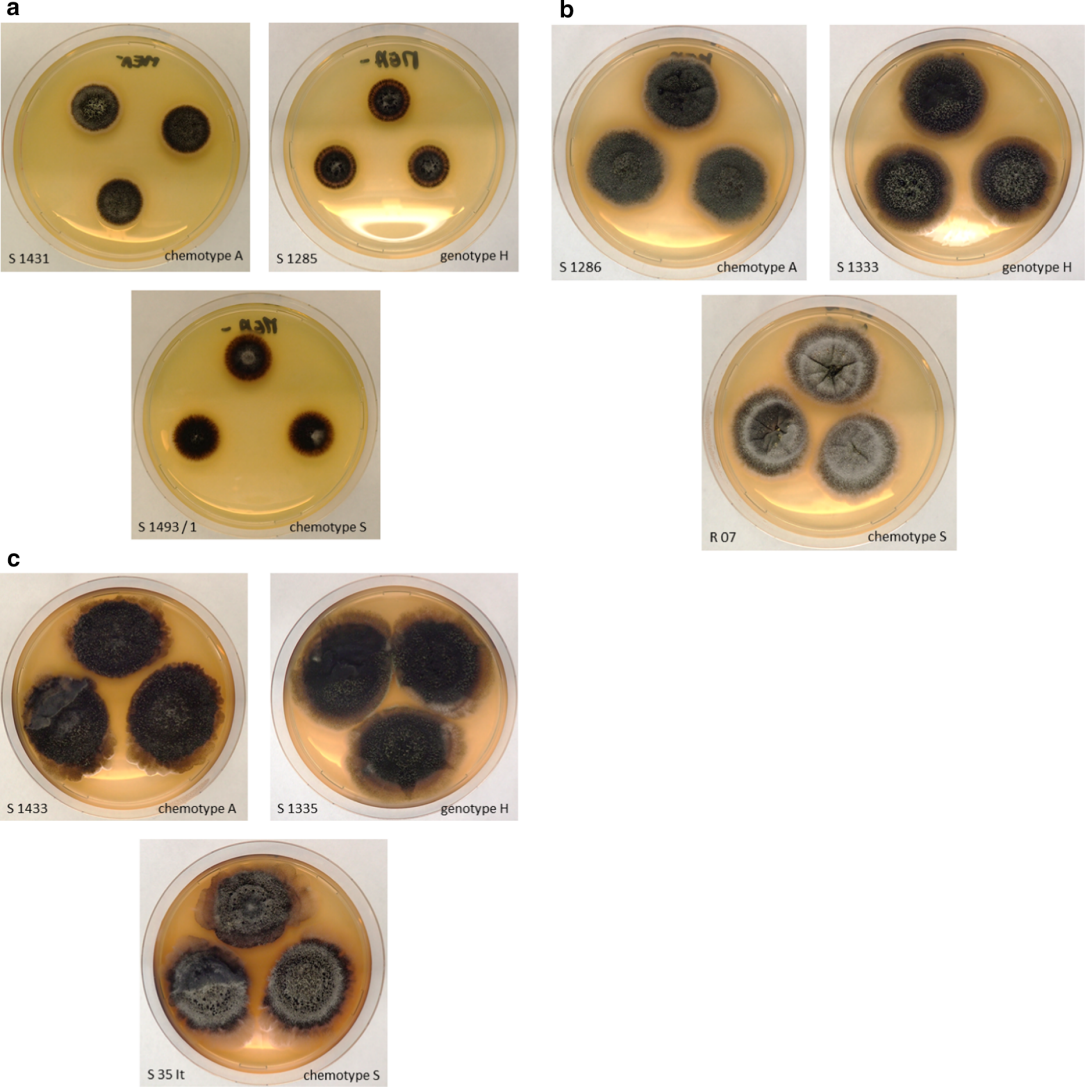
*S. chartarum* isolates incubated on MEA for **a** 7 days, **b** 14 days, **c** 21 days

Recently, a new nomenclature for *S. chartarum* was introduced. *S. chartarum* isolates can be distinguished into three genotypes based on distinct gene clusters (atranone- and satratoxin-gene clusters). The newly introduced hybrid genotype H contains the complete gene set for atranone production but an incomplete set for macrocyclic trichothecenes and the first toxin analysis revealed no detectable macrocyclic trichothecenes [19]. Every strain has been cross-checked via PCR analysis based on the proposed new nomenclature. The results are summarized in Table 1 and the actual visualization of the DNA fragments can be seen in Fig. 1. The size of the DNA fragments turned out as expected and each isolate could be classified as one of the two distinct chemotypes, the third genotype H, respectively (S-type: 346 bp, A-type: 230 bp, H-type: both fragment sizes). In Fig. 2, representative cultures of the chemotypes and the hybrid genotype H are displayed. Neither the acknowledged chemotypes A and S nor the recently described genotype H possess phenotypic characteristics that enable a reliable distinction.

### FT-IR Spectroscopy

**Fig. 3:**
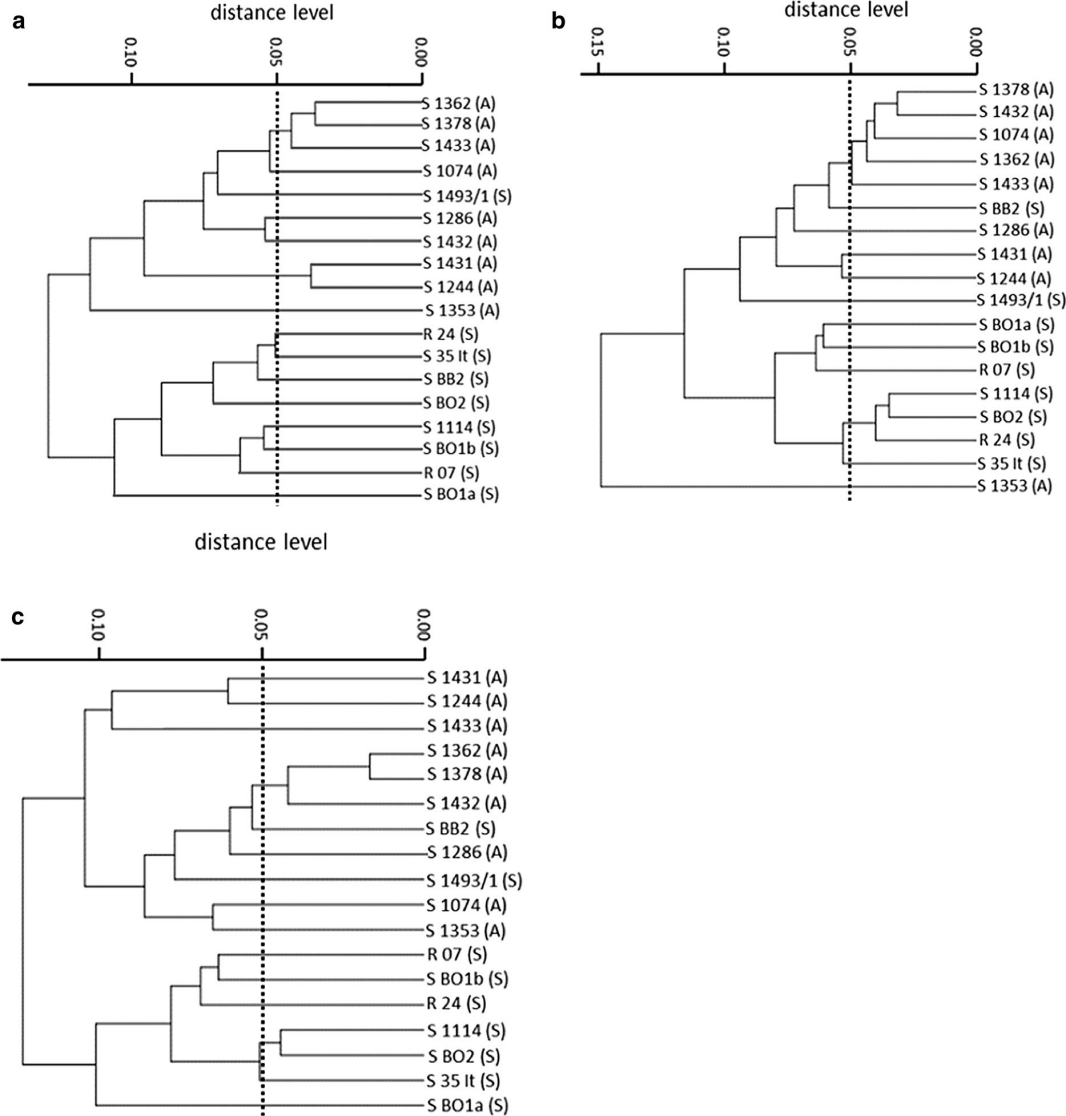
Dendrogram of S. chartarum strains measured by FT-IR spectroscopy comparing the chemotypes A and S incubated on MEA; (A) = A-type, (S) = S-type; dotted line = suggested cut-off value, **a** incubation for 7 days, Kappa = 0.89; *p* < 0.01, **b** incubation for 14 days, Kappa = 0.67; *p* < 0.01 and **c** incubation for 21 days, Kappa = 0.78; *p* < 0.01

For the differentiation of the two chemotypes A and S, a classification in two distinct clusters was obtained after 7 days of incubation on MEA. All but one isolate (S 1493/1) were correctly classified as chemotype A or chemotype S. The Cohen’s Kappa value (= 0.89; *p* < 0.01) shows that the accordance in this case is “almost perfect” according to the categories of Kappa values (Fig. 2a) [29, 30]. The prolongation of the incubation period resulted in a less correct differentiation and lower Cohen’s Kappa values (Fig. 3b, c). The Cohen’s Kappa values decrease to 0.67 (day 14) and 0.78 (day 21; both “substantial accordance” [29]). Noticeably, the isolate S 1493/1 remains in the chemotype A cluster after 14 and 21 days of incubation. Furthermore, after being incubated on MEA for 14 and 21 days, the isolate S BB2 is shifted from the chemotype S cluster to the chemotype A cluster. Besides, the strain S 1353 is correctly classified as chemotype A in Fig. 2a, c. Contrarily, after 14 days of incubation, it is neither assigned to the chemotype A cluster nor to the genotype S cluster.

Due to the fact that cut-off values for the differentiation on the strain level are only defined for bacteria or yeasts, experimental cut-off values for the distinction of chemotypes were suggested [28]. Regarding the results for the incubation on MEA, the lowest suggested cut-off values are 0.05 in all three cases. Overall, the distinction levels fluctuate, but a distinction appears to be reliable, if the cut-off value is between 0.1 and 0.05. In comparison to cut-off values for the distinction of bacterial strains, the suggested values in this study are clearly lower [28]. To state an example, analysing *P. Aeruginosa* with the basic IR-extraction and the settings used in the study at hand, the recommended cut-off values for a reliable distinction lie between 0.2 and 0.25 [28]. These low cut-off values for *S. chartarum* chemotypes result from the fact that only isolates of one species were analysed. The evaluation of the analyses aimed at the differentiation of *S. chartarum* strains on the subspecies level, chemotypes precisely.

**Fig. 4:**
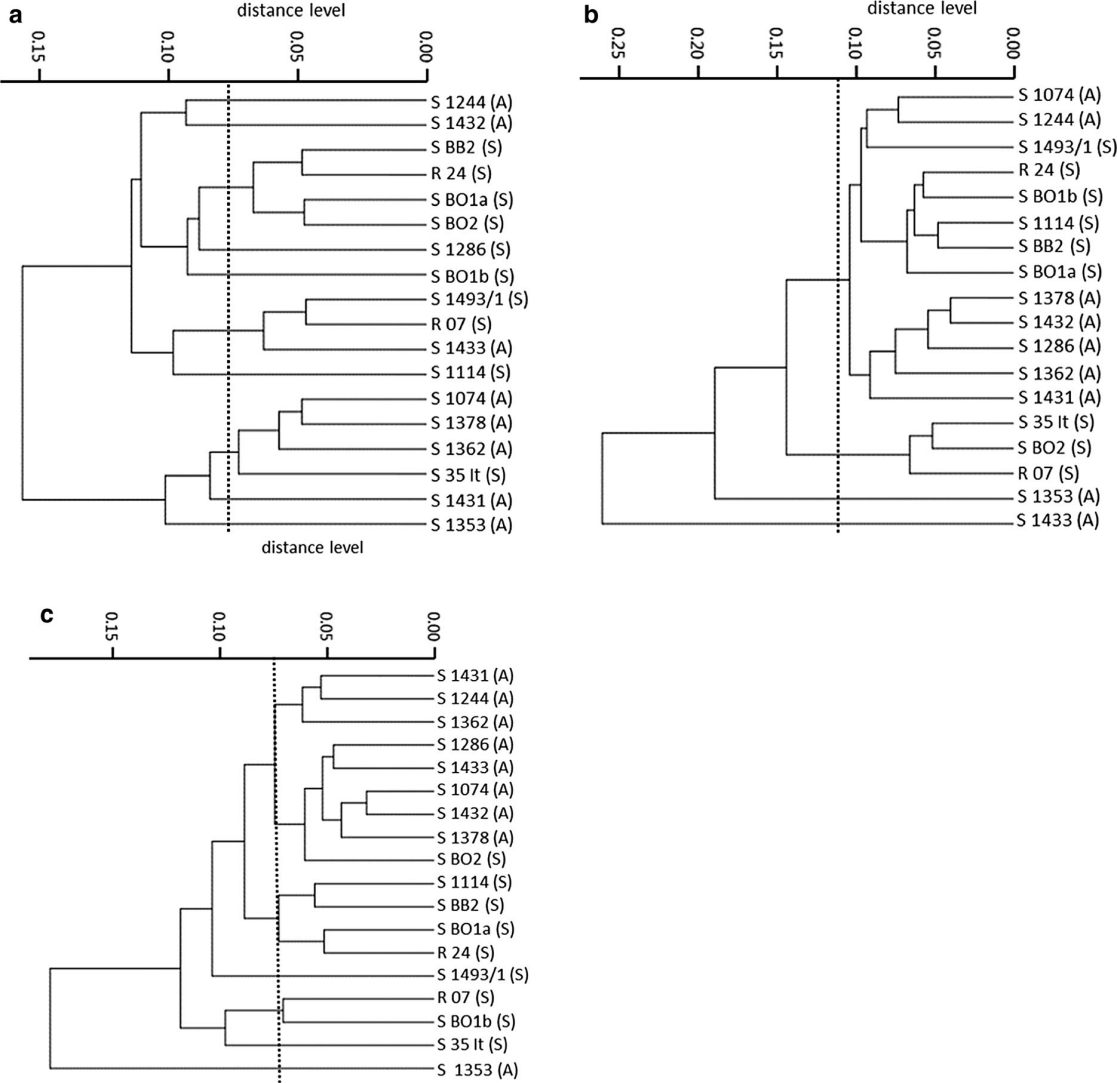
Dendrograms of *S. chartarum* strains measured by FT-IR spectroscopy comparing the chemotypes A and S incubated on PDA; (A) = A-type, (S) = S-type; dotted line = suggested cut-off value, **a** incubation for 7 days, Kappa = 0.44; *p* = 0.05, **b** incubation for 14 days, Kappa = 0.11; *p* > 0.5 and **c** incubation for 21 days, Kappa = 0.22; *p* < 0.5

The results for the cultivation on PDA generally resulted in low Cohen’s Kappa values (highest value on day 7: 0.44; “fair accordance” [29]). Furthermore, two clusters are only distinguishable after incubation for 7 days. The correct characterization of the two chemotypes is only “moderate” (0.44, Fig. 4a) [29]. After further incubation for 14 and 21 days, even no main clusters are discernible (Fig. 4b, c). Consequently, no reliable characterization was possible after cultivation of *S. chartarum* isolates on this type of agar.

The basic IR-extraction method had to be optimized in this study, because it is only adapted to bacteria and yeasts [28]. Thus, the basic IR-extraction protocol was replenished with further treatments. Avoiding sporulation and dark pigmentation, the usual incubation of *S. chartarum* on solid media was followed by an incubation period in liquid media [31]. The samples continued to grow for 4 days in liquid medium and provided enough biomass for technical replicates. Further washing steps with ultrapure water were included in the optimized protocol to avoid contamination of the sample material by residues of the medium. An additional homogenization step finally enabled the measurement of the fungal sample material.

The evaluation of the measurements revealed that the hybrid genotype H cannot be distinguished from the low-cytotoxic chemotype A or from the highly cytotoxic chemotype S via FT-IR spectroscopy (Table 2).

**Table 2:** Kappa and *p* values for *S. chartarum* strains measured by FT-IR spectroscopy comparing genotype H to the Chemotypes S and A

	Genotype H versus Chemotype S						Genotype H versus Chemotype A					
Medium	MEA			PDA			MEA			PDA		
Incubation period (days)	7	14	21	7	14	21	7	14	21	7	14	21
Kappa	0.35	0.68	0.57	0.27	0.24	0.57	0.24	0.37	0.43	0.08	< 0	0.09
*p*	> 0.05	< 0.01	< 0.05	> 0.05	> 0.05	< 0.01	> 0.05	> 0.05	0.05	> 0.05	< 0.01	> 0.05

*MEA* Malt extract agar, *PDA* Potato dextrose agar

## Discussion

The two chemotypes of *S. chartarum* could be distinguished after incubation on MEA for 7 days, all but one isolate (S 1493/1) were characterized as the correct chemotype. As the amount of toxins increases over time, the correct categorization of the chemotypes was expected to increase simultaneously [32]. Unexpectedly, our results revealed that the number of falsely categorized isolates increased (Fig. 3). The most conspicuous isolates of the incubation period on MEA are S 1493/1, S BB2 and S 1353. The strain S 1493/1 is wrongly classified as chemotype A in all three cases, although it was confirmed to produce macrocyclic trichothecenes in previous LC–MS/MS analyses. The ability to produce cytotoxic toxins might not be the relevant criterion for distinction. In addition, various subcultivations of the strains might lead to mutations which modify the molecular bonds and the unique spectral data [33]. Contrarily, the strains S BB2 and S 1353 are characterized unequivocally over time as different chemotypes (Fig. 4). According to Shapaval et al. (2010), the spectral regions “fatty acids + lipid” (3200–2800 cm^−1^, 1300–1000 cm^−1^) and “fingerprint” (900–700 cm^−1^) are the most suitable for the distinction of species in hierarchical cluster analysis [25]. The study at hand covers most of the suggested spectral regions. The relevant regions for the differentiation of *S. chartarum* on the subspecies level might lie beyond these regions. Moreover, the ambiguous characterization of these isolates over time might result from unrevealed differences or characteristics. As far as we are informed, only one other study about FT-IR spectroscopy aimed at the distinction of fungal strains in terms of toxin production. Kaya-Celiker et al. [34] successfully distinguished peanuts contaminated with non-toxic strains of *A. flavus* from those contaminated with toxic strains. The method used was not applicable for this study as a completely different FT-IR system was used. The phenomenon of isolates which are characterized differently below the species level has not been reported yet.

To evaluate the impact of the cultivation media, MEA and PDA were used because both media are commonly used in studies on the characterization of *Stachybotrys* spp. [11, 32, 35]. However, PDA turned out to be not suitable for the incubation of *S. chartarum* as already observed elsewhere [3].

As the treatment of fungal materials for FT-IR spectroscopic methods implies several difficulties, e.g. hydrophobic conidia, pigments or unstable biochemical characteristics of spores after the application of chemicals, various protocols for the sample preparation of different fungal species were developed [23, 25, 36]. The most applicable workflow included an additional incubation period in liquid medium and a homogenization step [25]. Thus, the amount of biomass was increased for technical replicates and the dark pigmentation was reduced. These optimizations finally enabled the application of FT-IR spectroscopic measurements to *S. chartarum*.

In this study, the subdivision of *S. chartarum* into three genotypes could not be confirmed via FT-IR spectroscopic measurements. However, regarding the results of the identification via PCR for satratoxin- and atranone-gene clusters, each strain could be identified as the expected chemotype or genotype. Above all, the previous differentiation of *S. chartarum* into a macrocyclic trichothecene type (chemotype S) and an atranone type (chemotype A) seems to be the most appropriate characterization when it comes to terms of FT-IR spectroscopic analysis [11].

To evaluate the health risk for humans, the reliable characterization of a *S. chartarum* isolate as chemotype S is very important as a first indication, but it does not allow a statement about the actual health risk. Exposure depends on a variety of factors such as the general ability to produce mycotoxins which is influenced by various external factors, the amount of mycotoxins produced and finally the way of exposure (direct contact, ingestion, inhalation) [37, 38]. The methods of choice for the detection and evaluation of the toxic potential of *S. chartarum* strains remain LC–MS/MS and cytotoxicity testing as the results of the FT-IR analysis can only be seen as an indication of chemotype characterization [39, 40]. After these first analyses, FT-IR spectroscopy seems to be too time-consuming with inconsistent results for the characterization of *S. chartarum* chemotypes in practical terms. In conclusion, the differentiation of the *S. chartarum* chemotypes via FT-IR spectroscopy can be achieved if the isolates are incubated for 7 days on MEA. It is not advisable to prolong the incubation period to increase reliability. For FT-IR spectroscopic measurements, the distinction of *S. chartarum* into an atranone- and a satratoxin-type remains the adequate differentiation.

## Electronic supplementary material

Below is the link to the electronic supplementary material.

Supplementary file1 (DOCX 120 kb)

## Abbreviations

FT-IR: Fourier-transform-infrared spectroscopy
LC–MS/MS: Liquid chromatography–mass spectrometry
MALDI–TOF MS: Matrix-assisted laser desorption/ionization–time of flight mass spectrometry
MEA: Malt extract agar
MEB: Malt extract broth
PDA: Potato dextrose agar
PDB: Potato dextrose broth
*S.*: *Stachybotrys*
