# Supplementary material for: Differentiation of S. chartarum (Ehrenb.) S. Hughes Chemotypes A and S via FT-IR Spectroscopy
Source: Mycopathologia. 2020 Oct 10;185(6):993–1004. doi: 10.1007/s11046-020-00495-0 (PMC7779419; doi:10.1007/s11046-020-00495-0)
Supplement: Supplementary file 1 — Supplementary file1 (DOCX 120 kb) [file 11046_2020_495_MOESM1_ESM.docx]

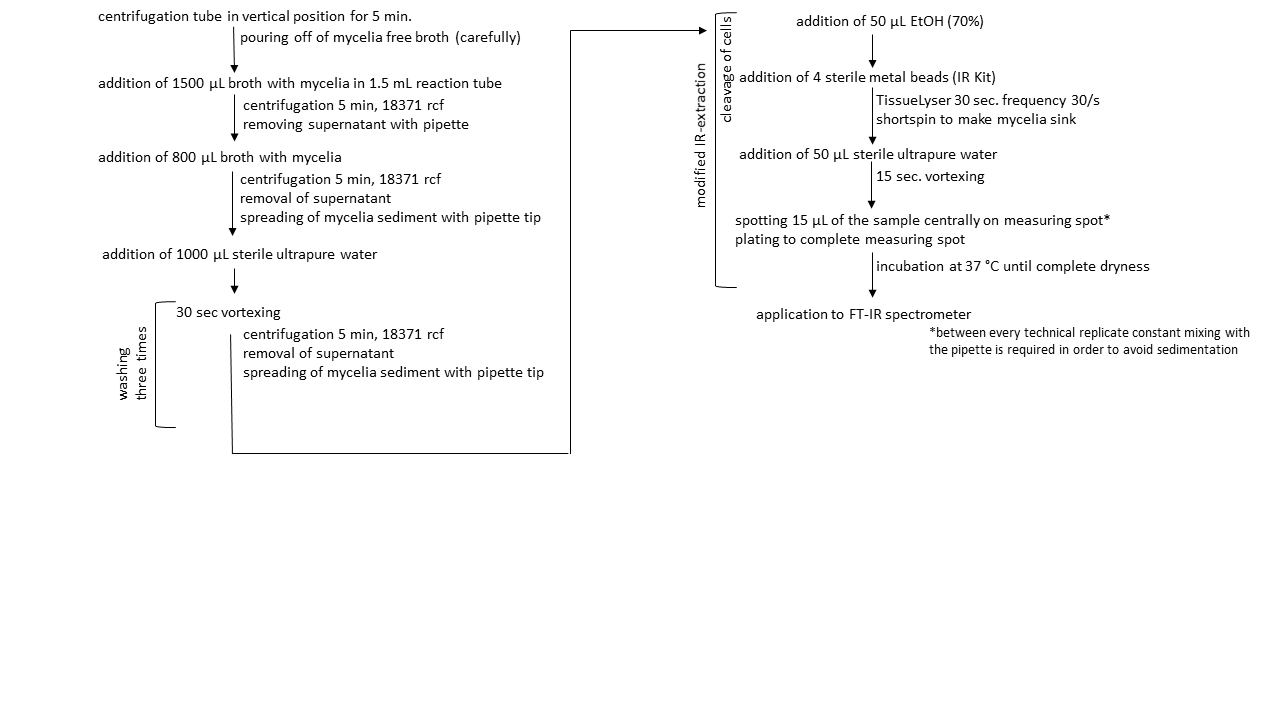


**Figure S1:** Workflow for the preparation of S. chartarum isolates for application to FT-IR measurements
